# Innovations in Thoracic Surgery and Medicine—From Concepts to Real-World Care †

**DOI:** 10.3390/life15121874

**Published:** 2025-12-08

**Authors:** Paolo Scanagatta

**Affiliations:** Division of Thoracic Surgery, Morelli Hospital, ASST Valtellina e Alto Lario, 23035 Sondalo, Italy; paolo.scanagatta@asst-val.it; Tel.: +39-0342808804; Fax: +39-0342808616

## 1. Introduction and Scope

Thoracic surgery and respiratory medicine are transitioning from device-centric innovation to systems-level transformation. Robotic platforms, image-derived biomarkers, and artificial intelligence (AI) are maturing alongside standardized perioperative pathways and increasingly precise systemic therapies. However, genuine progress depends on whether these advances can be integrated into routine care that contends with geographic disparities, workforce constraints, budgetary pressures, and the imperative of environmental sustainability. In other words, innovation matters only insofar as it is implementable, auditable, and equitable at the bedside.

The aim of this Special Issue was to document solutions that close the “last mile”—the gap between promising methods and real-world benefit. We sought contributions that paired technical rigor with practical delivery; for example, pathway redesign, deployable analytics, and clinical decision support systems that can be monitored and improved over time within a learning health system (LHS) [1].

The three main messages conveyed across the published papers were as follows:

- Workflow engineering delivers immediate value. Well-designed service models—such as fast-track diagnostic routes, protected procedure/diagnostic slots, teleconferenced multidisciplinary team (MDT) meetings, and proactive case management—can reduce avoidable delays and variation even before new technologies are introduced.
- Trustworthy analytics are non-negotiable. Imaging and AI tools must report calibrated performance, undergo external validation, and follow prespecified analyses. Data and code should adhere to FAIR principles (Findable, Accessible, Interoperable, Reusable) to support reproducibility and safe clinical translation [2,3,4,5].
- Ensuring equity and implementation must be part of the design process. Innovations should narrow—rather than widen—gaps in access, timeliness, and outcomes. Evaluations therefore require operational endpoints (time to diagnosis/treatment, avoidable procedures), patient-reported outcomes, and cost considerations across diverse settings [6,7,8].

The sections that follow summarize each contribution and extract practical lessons, then outline priorities for the confirmed Second Edition (2026), emphasizing multicenter implementation studies, externally validated clinical analytics, and scalable workflow models that make innovation work for all patients.

## 2. An Overview of the Published Articles

Below, we briefly summarize the five contributions that comprise this Special Issue, emphasizing what is currently implementable, what is still in need of external validation, and what can be scaled across diverse care settings.

For each paper, we describe the clinical task performed, the study design, the key take-home message, and the relevance to day-to-day care.

### 2.1. Fast-Track Diagnostics in a Complex Setting (Original Research)

Scanagatta et al.’s [Contribution 1] pathway study demonstrates how service redesign can compress time to diagnosis and reduce unwarranted variation despite organizational complexity. Key levers included protected diagnostic slots, early nurse-led case management, structured triage to multidisciplinary team (MDT) review, and proactive escalation rules. Beyond traditional clinical outcomes, the authors report operational endpoints (e.g., median days to tissue diagnosis and to first treatment, avoidable repeat procedures), making the model auditable and transferable [1]. Their work exemplifies how workflow engineering can deliver near-term benefit while creating the infrastructure needed for later AI or decision-support integration [8].

### 2.2. Imaging Complexity Biomarkers to Predict Symptomatic Radiation Pneumonitis (Original Research)

Hwang et al. [Contribution 2] developed and tested computed tomography (CT)-derived “complexity” descriptors of parenchymal disease to anticipate clinically significant radiation pneumonitis in non-small cell lung cancer [9,10]. By focusing on comorbidity-informed imaging signals rather than tumor morphology alone, their study addresses a high-value clinical consideration (treatment toxicity). The authors describe the model’s calibration and cross-validation, paying close attention to clinical interpretability and the ways in which such biomarkers might be incorporated into planning workflows [2]. Their study illustrates the path from technically feasible features to bedside-relevant risk stratification aligned with trustworthy analytics principles [3].

### 2.3. Artificial Intelligence and Lung Cancer Screening (Narrative Review)

The review by Duranti et al. [Contribution 3] synthesizes the rapidly evolving interface between population screening and AI across eligibility optimization, nodule detection/characterization, growth assessment, and program operations [6,7]. It emphasizes where evidence is already actionable (e.g., reader-assisted triage and quality control), where prospective validation is still needed (e.g., fully automated management recommendations), and how FAIR data and external validation will determine generalizability. Importantly, the authors discuss implementation guardrails—calibration drift monitoring, human-in-the-loop checks, and equity-aware performance auditing—to ensure that automation improves, rather than fragments, screening pathways [6,11].

### 2.4. Pulmonary Adenocarcinoma in Rheumatoid Arthritis: A Case Report with Literature Synthesis

Constantin et al. [Contribution 4] examined a diagnostically challenging scenario—lung cancer arising in the context of rheumatoid arthritis and its therapies [12]. The case summary is coupled with a concise literature review on risk modification, imaging pitfalls (e.g., inflammatory confounders), and peri-treatment considerations [13]. The educational value lies in practical MDT implications, such as when to escalate from surveillance to tissue sampling, how to weigh immunosuppression, and how to communicate risk with patients with overlapping inflammatory and oncologic burdens.

### 2.5. Metastatic Malignant Peripheral Nerve Sheath Tumor (MPNST): Surgery Plus Targeted Therapy (A Case Report with a Literature Review)

In their paper, Skórka et al. [Contribution 5] address a rare, aggressive entity that is frequently linked to neurofibromatosis type 1 [14]. Their report details a surgical strategy for locally advanced/metastatic disease and the rationale for adjunct targeted agents, highlighting toxicity management and functional outcomes in a young adult. The literature synthesis clarifies what is known about molecular drivers and when systemic therapy might complement resection. Although uncommon, such cases pressure-test MDT coordination, survivorship planning, and access to precision oncology, which are all core themes of this Special Issue.

#### Cross-Cutting Take-Aways

Together, these articles outline a coherent pathway from service redesign to trustworthy analytics, setting the stage for the priorities detailed in Section 3.

Across the five papers, three messages recur:

(i) Process matters—measurable pathway redesign can yield rapid gains and help prepare the ground for digital tools.
(ii) Analytics must be trustworthy—feature engineering and models should report calibration and undergo external validation prior to clinical adoption.
(iii) Implementation and equity are part of the evidence—case-based syntheses expose real-world constraints and help teams standardize decisions regarding complex or rare scenarios.

These lessons directly inform the key priorities to be addressed in the Second Edition (Section 3).

## 3. Perspective for the Second Edition (2026)

The Second Edition will share this Special Issue’s emphasis on implementable, auditable, equitable innovation [1], but will ask authors to go one step further, demonstrating measurable impact in real-world settings and reporting their findings in ways that make replication straightforward. The priorities and concrete expectations of the upcoming Second Edition are listed below.

### 3.1. Pragmatic Pathway Trials and Auditability

We welcome multicenter evaluations of fast-track diagnostic and treatment pathways with harmonized indicators and pre-registered analysis plans. At minimum, we aim to gather contributions that address the following topics:

- Timeliness: median and 90th percentile time to first specialist review, tissue diagnosis, staging completion, and treatment start, as well as the proportion of patients starting treatment within guideline windows.
- Effectiveness: stage distribution at diagnosis; resection or radical therapy rates; unplanned admissions; and avoidable repeat procedures.
- Patient-centered outcomes: patient-reported outcome measures (PROMs) and experience measures (PREMs); communication quality; and travel/time burden.
- Safety and resources: procedure-related complications; staff time and pathway costs (from both payer and societal perspectives) with sensitivity analyses.
- Equity: results stratified by geography, age, sex, socioeconomic indicators, and comorbidity.

Preferred designs include stepped-wedge or before–after designs with concurrent controls when cluster randomization is impractical. Analyses should adjust for secular trends and seasonality and present effect sizes with 95% confidence intervals, not *p*-values alone [6,7,8].

### 3.2. Trustworthy Imaging and AI at the Point of Care

Submissions on imaging analytics and artificial intelligence (AI) should move from feature discovery to decision support. We encourage the following:

- External validation on truly independent datasets with patient-level splits (no frame-level leakage) for imaging.
- Full calibration reporting (calibration plots, calibration-in-the-large, slope) and prediction intervals, together with discrimination metrics.
- Decision-curve analysis to quantify clinical utility at actionable thresholds.
- Model documentation (model cards), code or containers when feasible, and dataset descriptors aligned with FAIR principles (Findable, Accessible, Interoperable, Reusable).
- Bias and subgroup analyses (age, sex, comorbidity; center/vendor effects), with plans for drift monitoring after deployment.

As minimum reporting standards, submissions should comply with TRIPOD (Transparent Reporting of a multivariable prediction model for Individual Prognosis Or Diagnosis) for prediction models, CONSORT-AI (Consolidated Standards of Reporting Trials–AI extension) for interventional trials of AI, and PRISMA-DTA (Preferred Reporting Items for Systematic Reviews and Meta-Analyses of Diagnostic Test Accuracy) for evidence syntheses, as appropriate [3,4,15]. Moreover, we suggest including calibration, external validation, decision-curve analysis, and FAIR-aligned code/data descriptors wherever feasible.

### 3.3. Screening Strategies That Blend Modalities and Reduce Harm

We are particularly interested in pragmatic studies that combine low-dose CT screening with AI triage and/or blood-based biomarkers, powered not only for area under the receiver operating characteristic curve (AUC) but for operational endpoints:

- False-positive cascades (avoidable invasive work-ups and cumulative radiation dose).
- Time from positive screening to treatment decision.
- Stage shift and curative-intent therapy rates.
- Cost-effectiveness and budget impact across different delivery models.
- Equity metrics: participation and outcomes in underserved or rural populations.

These studies should prospectively define management thresholds and include human-in-the-loop safeguards and calibration drift monitoring [6,7,8,11].

### 3.4. Rare Tumors and Complex Comorbidity

We seek collaborative registries and case–cohort series for rare thoracic entities (e.g., NF1-associated malignant peripheral nerve sheath tumor, thymic tumors, mesothelioma) and for oncology in the context of multimorbidity (e.g., autoimmune disease and interstitial lung disease) [14]. Priorities include the following:

- Harmonized minimum datasets (clinicopathologic, molecular, treatment intent, toxicity, and longitudinal outcomes).
- Molecular tumor board workflows and criteria for surgery within multimodality care.
- Decision tools that incorporate competing risks and patient preference, with transparent thresholds for escalation or de-escalation [13].

### 3.5. Learning Health-System (LHS) Infrastructure and Equity by Design

We invite reports that make improvement visible and durable, which include the following steps:

- Implementation of tele-multidisciplinary team (MDT) meetings, case management standards, and protected diagnostic slots across networks.
- Interoperable data layers that enable routine plan–do–study–act cycles, public dashboards, and benchmarking [1].
- Co-design with patients and staff; patient and public involvement (PPI) methods and outputs.
- Environmental metrics (e.g., travel reduction and resource utilization) where relevant. Equity analyses should be prespecified, with mitigation strategies when disparities are detected [5,6,7,14].

### 3.6. Requested Submissions for the Second Edition

- Original research: For the Second Edition of this Special Issue, we are seeking pragmatic pathway evaluations, works that include, externally validated imaging/AI tools with decision impact, and works which describe comparative effectiveness in screening and perioperative care.
- Method papers: We are interested in standard operating procedures, reporting templates, and open reference datasets that others can adopt.
- Evidence syntheses: We welcome systematic reviews and meta-analyses that set actionable thresholds for practice, reported per PRISMA-DTA or relevant extensions.
- Brief reports and case-based learning: Relevant submissions should highlight operational dilemmas (e.g., comorbidity trade-offs, rare tumor decision points) and include generalizable checklists.

## 4. Conclusions

- The Second Edition (2026) of this Special Issue will sharpen our focus on multicenter implementation, externally validated analytics, and scalable workflow models, ensuring that that promising ideas translate into better, fairer care for all patients. Concretely, our aims are as follows:
- Implementation at scale: We will prioritize pragmatic, multicenter studies that report a core outcome set (timeliness, stage shift, survival, patient-reported outcomes, equity, and costs) and utilize transparent analysis plans.
- Validation prior to deployment: We require external validation, calibration, and decision-curve analysis for imaging and artificial intelligence (AI) tools; share code and data descriptors should be aligned with FAIR principles (Findable, Accessible, Interoperable, Reusable).
- Design for equity and usability: Accepted studies will incorporate human-in-the-loop safeguards, bias audits, and drift monitoring. They will also measure access and outcomes across geography and demographics, with prespecified mitigation steps.
- Build learning health systems (LHS): We seek standardized fast-track pathways, tele-multidisciplinary team (MDT) practices, and protected diagnostic slots, as well as continuous plan–do–study–act cycles with interoperable data layers.
- We will favor changes to practice: The forthcoming Special Issue will prioritize work that delivers decision thresholds, checklists, and operating procedures suitable for immediate adoption.

In short, the 2026 edition will privilege impact you can measure, methods you can trust, and pathways you can scale, so that innovation moves from promising pilots to population-level benefit; from isolated excellence to equitable routine care.

## Data Availability

Not applicable.
